# The Influence of Interpreters’ Professional Background and Experience on the Interpretation of Multimodality Imaging of Pulmonary Lesions Using^ 18^F-3′-Deoxy-Fluorothymidine and ^18^F-Fluorodeoxyglucose PET/CT

**DOI:** 10.1371/journal.pone.0060104

**Published:** 2013-04-02

**Authors:** Bai-xuan Xu, Chang-bin Liu, Rui-min Wang, Ming-zhe Shao, Li-ping Fu, Yun-gang Li, Jia-he Tian

**Affiliations:** Department of Nuclear Medicine, Chinese PLA General Hospital, Beijing, People’s Republic of China; Wayne State University, United States of America

## Abstract

**Objective:**

Based on the results of a recently accomplished multicenter clinical trial for the incremental value of a dual-tracer (18F-FDG and 18F-FLT), dual-modality (PET and CT) imaging in the differential diagnosis of pulmonary lesions, we investigate some issues that might affect the image interpretation and result reporting.

**Methods:**

The images were read in two separate sessions. Firstly the images were read and reported by physician(s) of the imaging center on completion of each PET/CT scanning. By the end of MCCT, all images collected during the trial were re-read by a collective of readers in an isolated, blinded, and independent way.

**Results:**

One hundred sixty two patients successfully passed the data verification and entered into the final analysis. The primary reporting result showed adding 18F-FDG image information did not change the clinical performance much in sensitivity, specifity and accuracy, but the ratio between SUVFLT and SUVFDG did help the differentiation efficacy among the three subgroups of patients. The collective reviewing result showed the diagnostic achievement varied with reading strategies. ANOVA indicated significant differences among ^18^F-FDG, ^18^F- FLT in SUV (F = 14.239, p = 0.004). CT had almost the same diagnostic performance as 18F-FLT. When the 18F-FDG, 18F- FLT and CT images read in pair, both diagnostic sensitivity and specificity improved. The best diagnostic figures were obtained in full-modality strategy, when dual-tracer PET worked in combination with CT.

**Conclusions:**

With certain experience and training both radiologists and nuclear physicians are qualified to read and to achieve the similar diagnostic accuracy in PET/CT study. Making full use of modality combination and selecting right criteria seems more practical than professional back ground and personal experience in the new hybrid imaging technology, at least when novel tracer or application is concerned.

## Introduction

It has been known for years that the phonotype of many human diseases, in particular the malignant tumors, is based on a complex of sophisticated, internal-linked, multi-factor changes at molecular level [1]. Those molecular abnormalities, possessing the potential of diagnostic as well as therapeutic targets, are in fact beyond the scope of any single clinical detection technology developed so far. As the understanding of the complexity of biological processes in diseases deepened, many functional imaging modalities in addition to just morphological illustration, like CT, MRI or ultrasound, were employed to further enhance the clinical assessment of those diseases. Since the introduction and the early trials demonstrating its usefulness in 1998–2001, the hybrid imaging device, PET/CT, has induced a great change in the application and interpretation of medical imaging[2]–[5]. Ever since its value proved by the still expending clinical applications, PET/CT have gained a wide acceptance, even a separate codes assigned by the Centers for Medicare and Medicaid Services (CMS) allowing reimbursement for the increased cost of this technology in a big variety of tumors and other diseases [6]. In spite its advantages in more accurate morphological and metabolic data co registration, improved lesion localization, consolidation of PET and CT imaging studies, and reduced scan times, the hybrid imaging technique still had certain questions to be dealt with. For example, what is the best protocol and radiopharmaceutical(s) for tumor characterization with combined PET/CT imaging? Weather CT or PET should have the same weight in the hybrid-image interpretation? What qualification is needed for interpreting those images, or is radiologist and nuclear physician capable of reading images from opposite modality? There are also many other logistic and personal issues involved in the novel imaging modality. The current study was in fact a re-analysis based on the results of a recently accomplished multicenter clinical trial for the incremental value of a dual-tracer (^18^F-FDG and ^18^F-FLT), dual-modality(PET and CT) imaging in the differential diagnosis of pulmonary lesions [7], in which several radiologists and nuclear physicians actively joined, thus providing us the opportunity to investigate some issues that might affect the image interpretation and result reporting, such as (a)the consistency in reading multi-modality images by different readers and in different conditions, (b)the influence of the personal issues, like professional background and working experience of the readers, in the interpretation of the multi-modality images, and (c)the feasibility, objectiveness and readiness of the criteria chosen for the interpretation of those images.

## Materials and Methods

### The Clinical Trial

The multicenter clinical trial (MCCT) was designed according to the Good Clinical Practice (GCP) principles with the main goal of assessing the diagnostic performance of a dual-tracer PET/CT imaging for pulmonary nodules. This clinical trial was registered in PLA General Hospital with series number of L3012010057 (B), The Ethics Committee of Chinese People’s Liberation Army (PLA) General Hospital approved this study, and all patients signed written informed consent form. All works were undertaken following the provisions of the Declaration of Helsinki.

Twelve medical imaging centers (six from the Sourth and six from the North) located over a wide geographic area in China took parts in this randomized, blinded, prospective MCCT. One center acted as the organizer, taking the responsibility of conducting, supervising, collecting, verifying and final analysis of data, but isolated from clinical imaging. In the other 11 centers, patients underwent both ^18^F-FDG and ^18^F-FLT PET/CT scanning according the standard operation protocols (SOP) of the trial. The studied population consisted of patients of pulmonary nodule(s) with difficulty in defining the diagnosis by ordinary clinical means. Precautions were taken to ensure the integrity and originality of the data in acquisition, recording and collection. The images were read twice, and the results of each reading were compared to the standard of truth and comparatively analyzed for diagnostic efficacy. The pathological diagnosis, obtained via surgical processes or clinical evidence through therapeutic responses or lack of morphological change in follow-up for at least 12 months, served as the standard of truth. The primary reading was carried out in each imaging center immediately after imaging session, which provides clinicians a report and serves as the basis for management planning. The second reading by a collective of readers at completion of the trial offered the basis of investigating diagnostic performance of each reader in light of his/her professional background and experience. To ensure the compliance to the SOPs and the consistency of interpretation, the MCCT protocols were well discussed among the designing group and imaging centers. Trainings were provided to all participants as part of the preparation before initiation of the trial on data handling, standard of operation, and diagnostic criteria. The study protocol had been proven by the regional or hospital medical ethic committees, and a written consensus from every patient was obtained before the study.

### Study Protocols

All subjects underwent 2 PET/CT scans using ^18^F-FDG and ^18^F-FLT separately within 7d. The order of the radiopharmaceutical used was randomized. The radio tracers were synthesized by each imaging center using the same models of cyclotron (MiniTrace, GE) and automatic synthesizer (TracerLab Fx_FN_, GE). The radiochemical purity of every preparation of either radiopharmaceutical was >95%. The recommended intravenously injected dosage for both ^18^F-FDG and ^18^F-FLT was 4∼5 MBq/kg and a standard 60 min post-injection rest allowed before PET/CT scanning. The images were acquired using a similar model of PET/CT scanner (Discovery ST, GE, Milwaukee, USA). The CT was equipped with 4, 8 or 16 rows of detector, the PET with BGO crystal of a 15.7 cm axial field width, and a spatial resolution of 4 mm FWHM at 1 cm away from the center. The CT images was acquired at 120 kV, 100∼250 mAs, 0.8 sec rotation, 1.25 mm collimation with no other thin-slice scanning or contrast enhancement, followed by PET imaging. PET acquisition was in 3D mode, 2.5 min/bed, 4∼7 table position covering the entire chest or track of body. The images were reconstructed in FORE-Iterative algorithm, with post filter 4.7, loop Filter 3.8, subset 32, iteration 4, and matrix of 128×128. The mean CT value and the maximum standard uptake values (SUV_FDG_ and SUV_FLT_) were assessed via ROI set over the entire lesion volume. In case of more than one lesion, the maximum value or the biggest lesion was chosen as the representative. Images were primarily interpreted by each imaging center where the subject was imaged. The clinical management plan for each subject was obtained via a fill-out inquiry form from the referring clinicians before and after each imaging session. All the image data and recording sheets were collected and verified by the organizer. If question existed on the execution of the trial, the quality of either radiotracer or PET/CT scan, or doubt on the diagnosis by the end of follow-up, the data of the subject was rejected from further analysis. The operation and compliance to the trial protocols were under supervision and inspection of the organizer.

### The Image Reading Sessions

**Figure 1:**
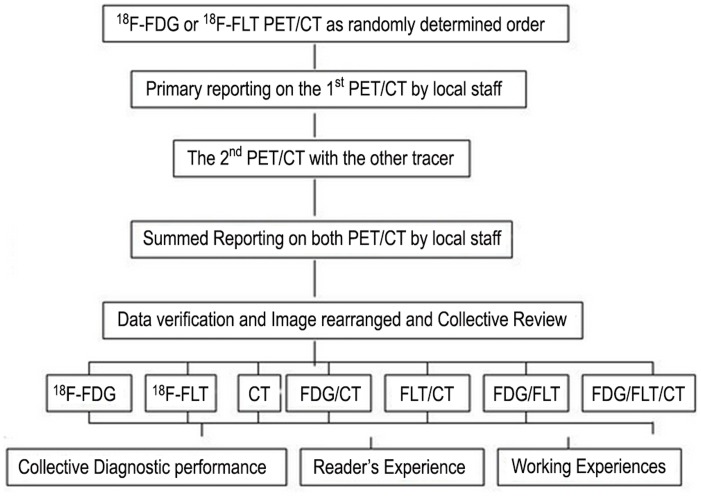
Schematic of primary and final collective image reading sessions. The diagnostic efficacy was assessed in light of the standard of truth. The primary report was carried out by local physicians in each imaging center. The collective image interpretation was carried out on completion of the trial with all images rearranged and blinded.

The images were read in two separate sessions (Fig. 1). Firstly the images were read and reported by physician(s) of the imaging center on completion of each PET/CT scanning. In this primary image reporting, the readers were provided with all the available data of the examinee. To standardize the image interpretation, every one responsible for the reporting was asked to follow the same criteria recommended by the organizer. The diagnostic features of all imaging centers were then summed and analyzed along with the clinical decision changes induced by the primary PET/CT reports.

By the end of MCCT, all images collected during the trial were re-read by a collective of readers in an isolated, blinded, and independent way. Sixteen readers (divided into two groups, eight from the North and eight from the South) took parts in the collective reading. Seven readers had professional background of CT, with working experience of 1∼20 years. The other nine were certified nuclear medicine physicians with 1∼26 years working experience. The readers had almost the same working experience with hybrid PET/CT imaging and with very limited experience of ^18^F-FLT image interpretation when the MCCT began. The personal features of the readers were listed in Table 1. In the final reading session, the patient’s images were rearranged into 7 sets for different interpretation strategies, i.e. single modality of ^18^F-FDG PET, ^18^F-FLT PET and CT, dual-modality of ^18^F-FDG+^18^F-FLT, ^18^F-FDG+CT, ^18^F-FLT+CT, and full modality of ^18^F-FDG+^18^F-FLT+CT. The presentation order of the subjects’ images for each interpretation strategy was randomized with all the readers unaware of information related to the subject. Each reader worked independently on a sheet marked only with his/her code number, making his/her own judgment for each patient as malignant, benign or undetermined. The working sheet of every reader was collected before the next round of reading. The collective diagnosis for any individual subject was determined by a consensus reached by over 8/16 readers. The diagnostic performance of each reader, and that achieved in consensus, were compared and analyzed in light of their personal features.

**Table 1:** The personal issues of the readers from north and south.

	North			South		
	Profession	Experience*	PET/CT Work*	Profession	Experience*	PET/CT Work*
1	CT	3	2	NM	1	4
2	CT	7	5	NM		3
3	NM	26	6	NM	1	2
4	CT	10	5	NM		5
5	NM	4	4	CT	15	5
6	CT	5	9	NM	20	
7	NM	2	2	CT	1	2
8	NM	18	4	CT	20	4
AVE	NM/CT	12.5/6.3	4.6	NM/CT	10.5/5.3	3.1

Abbreviation: NM, nuclear medicine physician; CT, radiologist working with CT; AV, The average working years.

* : working experience in years.

In both primary and final reading, the same diagnostic criteria were utilized. The morphologic features of CT were carefully evaluated for malignant signs, e.g. the nodule(s) of soft-tissue density, speculated and notch on margin, and/or plural contraction, etc. Any radio tracer accumulation over the lung lesion(s) on PET images was visually evaluated and quantitative data as SUV was assessed. Malignancy should be suspected if a lesion with 3 or more morphological features recognized in CT, or SUV_FDG_ >2.5, SUV_FLT_ >1.35, and uptake of ^18^F-FLT somewhat lower than that of ^18^F-FDG. The SUV_FLT_/SUV_FDG_ ratio was also calculated in the final collective reading session.

### Statistical Analysis

On completion of the collective reading, the results were un-blinded. The significance of different image interpretation strategy, the influence of interpreters’ professional background and working experience based on the odds of correct-incorrect interpretation, and the difference of diagnostic performance were compared using Person Chi-square test. A commercial (SPSS11.0) and a dedicated (MINITABLE, GE, for 6 Sigma, GE) software packages were used for statistical analysis. The analysis was carried out by a group of independent staff. A statistician took active part in the design, data verification and final analysis for this trial.

## Results

### Multicenter Clinical Trial

The clinical trial was carried out from January 2006 to June 2010. The overall compliance to the SOPs and protocols by all imaging centers were satisfactory. One hundred sixty two patients successfully passed the data verification and entered into the final analysis. The studied population in Table 2, 86 males and 76 females, aging 44–82 yr, consisted of 87 lung cancers (39 lung adenocarcinomas, 20 lung squamous cell carcinomas, 10 bronchioloalveolar carcinomas, 3 large cell alveolar cell carcinomas, 2 small cell alveolar cell carcinomas and 13 others), 27 tuberculosis(TB), and 48 other benign lesions(inflammation, pseudo tumor, granulomatous and fibrotic lesions). No side effect was ever reported with either radiopharmaceutical or in PET/CT scanning. On completion of the trial, no matter which radiopharmaceutical was used first, an accurate diagnosis was worked out by the multi-modality imaging in 118/162 subjects (72.5%). A substantial change on clinical management was observed in subjects after the imaging, either from aggressive treatment to conservative means or vice versa, and a partial change in another, as prolonged observation or anti-inflammation. The majority of PET/CT induced alteration was proven correct in later clinical practice except in 7 (two tumor was misdiagnosed as TB, and 5 benign lesions tumors). The details of this MCCT was reported elsewhere [7].

**Table 2:** 162 Clinical cases grouping and FDG, FLT SUV results.

Patient group		N	SUV FDG (Ave±SD)	SUV FLT (Ave±SD)
Benign pulmonary lesion	Lung adenomas	32	2.42±1.28	1.06±0.72
	Tuberculosis	29	5.72±3.30	1.90±0.93
	Lung tumor-like lesions	14	4.59±2.03	1.96±1.09
	Total	75	4.1±2.79	1.54±0.97
Lung cancer	Lung adenocarcinoma	39	8.15±4.50	3.96±2.30
	Lung squamous cell carcinoma	20	9.79±4.53	4.46±2.03
	Bronchioloalveolar carcinoma	10	8.34±3.60	2.80±1.36
	Other tumors	18	9.00±5.00	4.10±1.89
	Total	87	8.72±4.51	3.97±2.11

### The Primary Reporting

As defined by the study protocol, the PET/CT images of each subject were initially interpreted by the corresponding physician(s) at the medical center where the imaging took place. The primary image interpretation was carried out with full knowledge of available information related to the studied subject. For the 162 patients undergoing ^18^F-FDG imaging first the summed Se, Sp and Ac were 10/11, 14/24, and 24/35. The false positive results reduced from 10 to 4, but false negative increased from 1 to 3 when the information of ^18^F-FLT imaging from the second PET/CT scan was added. For the 20 patients undergoing ^18^F-FLT scan first, the summed Se, Sp and Ac were 4/5, 9/15, and 13/20. Adding ^18^F-FDG image information did not change the clinical performance much (3/5, 10/15, and 13/20, respectively), but the ratio between SUV_FLT_ and SUV_FDG_ did help the differentiation efficacy among the three subgroups of patients. The summed sensitivity (Se), specificity (Sp) and accuracy (Ac) in the primary image reading were 13/16(81.25%), 29/39(74.36%), and 42/55(76.36%) respectively, lower than that in the collective reading. The diagnostic efficacy of the primary reading was summed in Table 3.

**Table 3:** Diagnostic Efficiency of the Primary ^18^F-FDG/^18^F-FLT PET/CT reading.

	N	Se	Sp	acc	PPV	NPV
Sum.Read	55	81.3%	74.4%	76.4%	56.5%	90.6%
FDG 1^st^	35	90.9%	58.3%	68.3%	50.0%	93.3%
FLT 1^st^	20	80.0%	60.0%	65.0%	40.0%	90.0%

Abbreviation: Sum.Read, the summed.

FDG 1^st^ : subjects underwent ^18^F-FDG PET first followed by ^18^F-FLT PET imaging.

FLT 1^st^ : subjects underwent ^18^F-FLT PET first followed by ^18^F-FDG PET imaging.

### The Collective Reviewing

**Figure 2:**
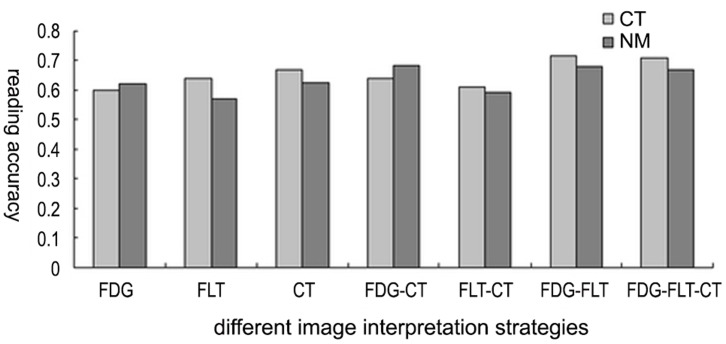
The comparison of the diagnostic performance of subgroups of readers. In most cases readers with CT background seems to have slightly better performance than those with nuclear medicine background.

**Figure 3:**
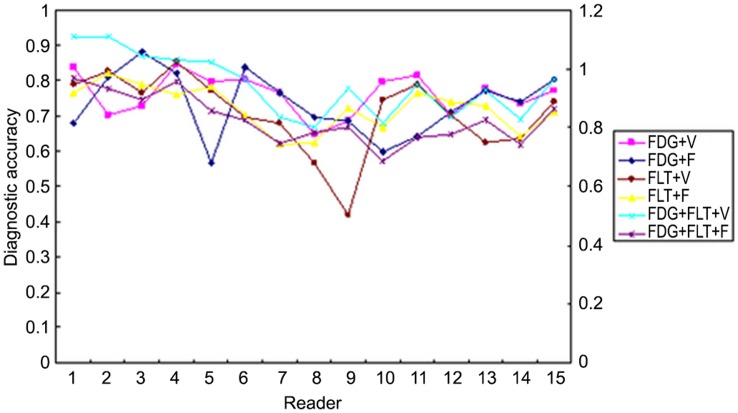
The variation of diagnostic accuracy in light of different reading strategies and readers. Although the diagnostic performance varied among readers, it seemed that all the stategies reading showed no difference in the variation of diagnostic accuracy.

It was found in the collective image reading that the diagnostic achievement varied with reading strategies (Table 4,). Significant differences existed between SUV_FDG_ of malignancies (8.72±4.51), TB (5.72±3.30) and other benign lesions (3.08±3.03) in ^18^F-FDG PET. ^18^F-FDG correctly detected 87 malignant lesions, but had false positive scans in 75 benign lesions. The detection rate of ^18^F-FLT PET was lower, so was the false positive rate (13/162). The uptake of ^18^F-FLT by the pulmonary lesion(s) was generally lower than that of ^18^F-FDG. The higher uptake by liver and bone marrow in vertebrae and ribs made the ^18^F-FLT images harder to read (Fig. 2). The SUV_FLT_ of malignancies, TB, and inflammation were 3.97±2.11, 1.90±0.93, and 1.33±0.83 respectively. ANOVA indicated significant differences among those subgroups in SUV (F = 14.239, p = 0.004). CT had almost the same diagnostic performance as ^18^F-FLT (TP 11/16, TN 29/39). CT helped the ROI drawing in cases of SPN smaller than 10 mm and in cases with very low uptake of ^18^F-FLT when reading became difficult. When the 3 images read in pair, both diagnostic sensitivity and specificity improved. The best diagnostic figures were obtained in full-modality strategy, when dual-tracer PET worked in combination with CT. The influence of different reading strategies on diagnostic performance was quite significant as Pearson Chi-square test indicated(*X^2^* = 16.725, *p* = 0.005). The ratio between SUV_FLT_ and SUV_FDG_ (FLT/FDG) was found useful in the separation of the 3 different subgroups of patients (Fig. 3). Setting a FLT/FDG window of 0.35∼0.90, 81/87 malignant tumors were correctly picked up, with only 6 false positive. Five tumors was false negative, with FLT/FDG ratio lower than 0.35. Among the rest of 75 lesions in this category, 27 were TB, and 48 inflammations. All lesions except one case with FLT/FDG over 0.90 were inflammation. The difference of FLT/FDG ratio among the 3 subgroups was significant (F = 3.927, *p* = 0.022).

**Table 4:** Diagnostic Efficacy of Image Interpretation Strategies in Collective Reading.

			Se	Sp	Acc	PPV	NPV
Single Modality	FDG PET		81.5%	59.0%	67.3%	46.7%	92.0%
	FLT PET		68.8%	76.9%	74.5%	55.0%	85.7%
	LDCT		68.8%	74.4%	72.7%	52.4%	85.3%
Paired Modality	FDG+CT	Visual	89.62%	44.76%	70.52%	68.84%	75.86%
		Film-Reading	84.49%	55.56%	71.66%	70.40%	74.35%
	FLT+CT	Visual	69.37%	54.79%	63.27%	67.40%	57.01%
		Film-Reading	73.65%	59.80%	67.14%	67.96%	66.39%
Full Multimodality	FDG+FLT+CT+PET	Visual	90.50%	59.89%	77.50%	75.46%	82.16%
		Film-Reading	88.13%	68.64%	79.57%	78.56%	81.75%

Abbreviation: Se, sensitivity; Sp, specifity; Acc, accuracy; PPV, positive predict value; NPV, negative predictive value.

FDG+FLT+CT: Full Multimodality.

### Influence of Profession Background

In the final collective image reading, as every reader worked in an isolated, self-working condition, complete blind to any information about the subject whose images he/she was reading, it offered an opportunity to compare the reader’s performance in light of his/her professional background. On completion of each image reading, the correct and wrong judgment of each reader in each reading session was counted and analyzed. It was found that the performance of each reader in all 7 interpretations did not relate to his/her professional background. Re-grouping the readers according to their professional background still found no difference in terms of the overall diagnostic performance between radiologists and nuclear physicians (*X^2^* = 1.668, *p* = 0.221, Fig. 2). The radiologists’ performances in each interpretation did not differ to that of nuclear medicine physicians (*p* = 0.394∼2.125). Carefully review of their diagnostic judgment, it was revealed that the readers with CT background seemed to be slightly more accurate than those with nuclear medicine in interpreting the images, especially CT, but the difference was not statistically significant(*X^2^* = 2.790, *p* = 0.076).

### Influence of Working Experience

**Figure 4:**
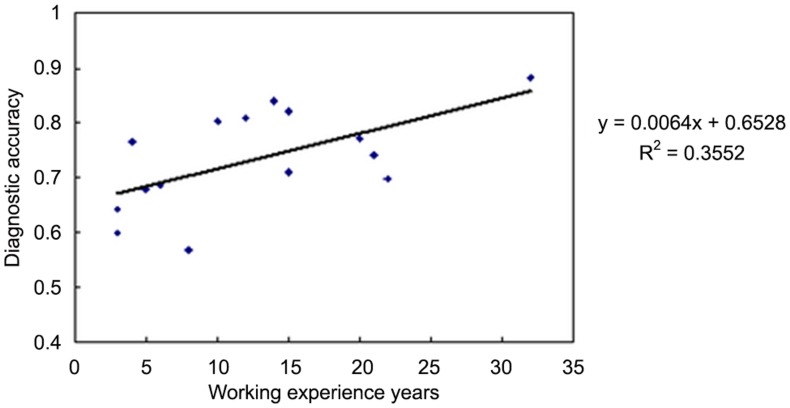
Correlation of the diagnostic performance and reader’s experience. A positvie correlation existed, and reach the significance, between reading accuracy and working years when 18F-FDG PET/CT was read.

In viewing the diagnostic performance of the readers in the collective review session, Chi square test indicated that the individual reader’s performance had no significant impact on the overall diagnostic figures(*X^2^* = 0.681, *p* = 0.731). In 6 interpretation strategies (except for the CT strategy), the performance of each reader varied unpredictably and showed no significant difference (Fig. 3). It was interesting, however, that a obvious trend of positive correlation, and also reached statistical significance, between the performance in interpretation of ^18^F-FDG PET/CT and working experience in years(*r^2^* = 0.355, P = 0.019, Fig. 4). In fact, the similar trend of correlation existed between reading performance and readers’ experience in all reading strategies but ^18^F-FDG+^18^F-FLT. It was also interesting that the variation among readers with different experience was much lower in the full-modality interpretation (^18^F-FDG+^18^F-FLT+CT) than other strategies. It could be noted as well from figure 4 that the readers with the minimum and the maximum working year had the lower variation in interpreting the images.

## Discussion

The introduction of a hybrid imaging device at the beginning of this century, merging together the morphological (CT) and metabolic (PET) information, has brought a significant change to the medical imaging technology. More and more authors has reported its incremental value than any single conventional imaging technique alone in diagnosing, staging, restaging and prognosis of a variety of tumors as well as of other non-tumor indications. This remarkable innovation offered metabolic information with morphological details, thus enhanced our understanding of many diseases, providing some insight into biological characteristics of a lesion. The hybrid device is so powerful, that its installation base worldwide increased dramatically in recent years, and it was assigned a CMS code and covered to certain extent by reimbursement system in many countries. Since the device consisted from two individual modalities, CT and PET, it has long been recognized that the responsibility of running the device required skill and knowledge of both radiology and nuclear medicine [8], [9]. In China, one third of about 100 scanners were installed in radiology, one third was run by nuclear medicine, 1/3 by radiology, and another 1/3 by independent party consisting of radiologists, physicians of nuclear medical and of other clinical specialties. An increasing number of authors in the literature addressed the importance of having both CT and nuclear medicine features in interpreting the hybrid images, emphasizing the complementary contribution of each technique [9], [10]. However, to the best of our knowledge, very few references could be found dealing with the influence of readers’ personal issues on the interpretation of the hybrid images so far, even though Blodgett et al emphasized the importance of logistic and personal issues in image interpretation and reporting [11], [12]. Thereafter, it was our main purpose to investigate if the professional background and working experience would affect the hybrid images interpretation, taking into our advantage that the design of the current MCCT, in which a collective radiologists and nuclear physicians read images in an isolated, independent, blind condition, provided an opportunity for analyzing the impact of those personal issues.

It was found that the best diagnostic efficacy could only be achieved through full utilization of the information derived from the dual-tracer (^18^F-FDG/^18^F-FLT) and dual-modality (PET/CT) imaging. The images obtained from ^18^F-FDG, ^18^F-FLT PET and CT could be readily and correctly interpreted by all readers at either on-spite primary reporting or in final collective reading. The diagnostic accuracy was not affected by either the professional background or working experience of the reader(s). In fact, no matter how the image data presented, in single modality, in pair, or in full combination, no significant influence could be detected in terms of the readers’ professional or experience in their performance in the interpretation. Looking at the correct-wrong number of each reader, the CT people and nuclear physicians almost had the similar reading accuracy, even when ^18^F-FDG and CT images were interpreted alone. The result was not entirely unexpected, because the enrollment criteria into the current study had precluded all subjects with typical radiological manifestation, i.e. all the subjects had pulmonary lesion(s) difficult to characterize by either radiological (CT) or functional (PET) means alone. Besides, all readers had almost similar working years with the hybrid device thus strongly indicated that the readers had learnt from the opposite discipline already and read the images in a similar way. It was noticed that, generally, CT doctors interpreted the images a little better than nuclear physicians, even though the difference was subtle and far from significance. Weather this reflect the higher weight of morphological information in those specially difficult subjects in our studied population, due to higher fraction of active TB and inflammation, which resulted in a dramatic higher false positive PET scan, needed further verification.

In analysis of the diagnostic performance in light of individual image reader’s experience, the results were a little surprising. The correct reading did not keep along with the working experience. Longer working experience did not mean better performance in the image interpretation. Searching through the literature, Ng et al once reported that an experienced nuclear physician did better than an inexperienced one in ^18^F-FDG but not in ^11^C-PIB to which none of the two had experience [13]. However, the comparison was only reported on two readers.

**Figure 5:**
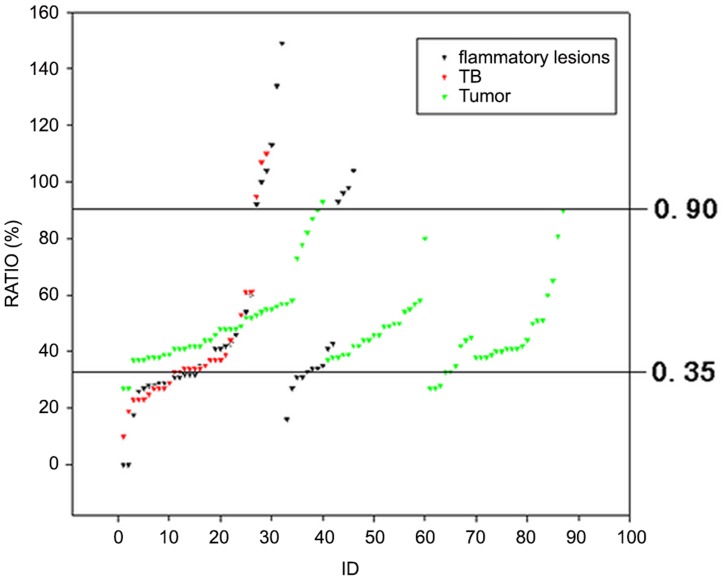
The separation of lung tumours, TB and other inflammatory lesions by SUVFLT to SUVFDG ratio. Most tumours were within a fixed ratio range of 0.35 to 0.90.

For FDG PET/CT strategy, the diagnostic accuracy showed significantly positive correlation with reader’s personnel work experience (Fig. 4). It could be interpreted as following: In our study, for all readers,^ 18^F-FLT was relatively new and special imaging agents. Most of the film reading diagnosis was conducted with less experience, and without mature diagnostic criteria. Therefore, the results “no significant correlation between the diagnostic accuracy and reader’s personnel work experience” could be well explained. However, compared with ^18^F-FLT, ^18^F-FDG was a mature, conventional cancer imaging agent, and there existed mature diagnostic criteria to be followed by readers. Therefore, with ^18^F-FDG PET/CT strategy, the longer PET/CT work experience meant the better diagnostic experience, and these readers could showed the better diagnostic accuracy in reading. For FDG PET/CT strategy, the diagnostic accuracy showed significantly positive correlation with reader’s personnel work experience (Fig. 5). It could be interpreted as following: In our study, for all readers,^ 18^F-FLT was relatively new and special imaging agents. Most of the film reading diagnosis was conducted with less experience, and without mature diagnostic criteria. Therefore, the results “no significant correlation between the diagnostic accuracy and reader’s personnel work experience” could be well explained. However, compared with ^18^F-FLT, ^18^F-FDG was a mature, conventional cancer imaging agent, and there existed mature diagnostic criteria to be followed by readers. Therefore, with ^18^F-FDG PET/CT strategy, the longer PET/CT work experience means the better diagnostic experience, and these readers could show the better diagnostic accuracy in reading.

The fact that CT people did slightly better than those with NM background in the current study, might also suggested shortage of the PET experience of CT staff, i.e. free of the influence of “old one’s” effect, partially accounted for their slightly better performance. Nonetheless, it had to be aware of that the importance of experience should never be neglected. The similar performance of radiologists and nuclear physicians in reviewing all images might resulted from something in common, that is the almost the same working time on the hybrid imaging modality of PET/CT despite their difference in professional background. Considering our goal in MCCT, on exclusion of the subjective influence as profession and experience, what we learned from this trial was that following the proper diagnostic protocols and interpretation criteria seems more reliable than any other personal issues, especially in cases of novel application in less experienced and clinical challenging situations.

In fact, the results of the final collective reading supported the overwhelming importance in diagnostic efficacy of proper interpretation strategy and criteria. Because of the atypical imaginary findings and the shortage of the related information, select the full-modality interpretation strategy guarantee the best diagnostic efficacy independent to either profession or experience of the reader(s). That fact, on the other hand, strongly suggested that the two disciplines involved in hybrid PET/CT actually needed each other. Many authors addressed the necessity of making use of CT features in PET/CT imaging [9]–[11], [13], and others claimed the PET should never be neglected [14]–[16]. In fact, from the result of the current study, the radiologists and nuclear physicians did not just depended on just their own discipline, but tried effectively accept the other’s view-points, just like Schoder et al commented [9]. It is important indeed to note that the correct diagnosis could only be obtained via not only the combination of CT and PET, but also the addition of the second radiotracer revealing more insight information of the lesions in the current study. In supporting the findings, a semi-quantitative index, the ratio of SUV_FLT_/SUV_FDG_ was found much more reliable and less variable in separating the subgroups of the lesions. Since the SUV ratio was assessed semi-automatically, its advantage of independence to any subjective judge was thus demonstrated. It was also obvious in our MCCT that the final efficacy could never be achieved if any of the imaging modalities missed in the interpretation.

It must be pointed out that the impressions drawn from the current study was still premature and care should be taken in considering it into other clinical settings. There were several “tricky” factors need to be addressed. First, the value of this study was more and less limited by the small numbers of patients as well as of the image readers. More significantly, the analysis was based on subjects of clinical challenging, i.e. patients with atypical clinical symptom, signs, laboratory and radiological manifestation. The diagnostic power of either PET or CT was thereby weakened. The situation we were facing in this MCCT could probably never present in the ordinary clinical practice. Second, no classical diagnostic CT was undertaken for imaging, thus the diagnostic potential and the real value of this most commonly used tool in pulmonary nodules might be underestimated. So could be the contribution of CT profession background in image interpretation underestimated. Third, since the majority of the readers had had no experience with ^18^F-FLT, the diagnostic and working experience seemed less meaningful in scenario. The readers lacked experience of the hybrid PET/CT imaging, because its recent availability. None of the readers had worked with the hybrid device experience longer than 4years when the clinical trial started. That means, no matter how long the reader had been working in his/her discipline, almost all readers had virtually the same experience with PET/CT when this trial started.

In conclusion, restrictive selection of subjects, standard operation and participation of readers from different disciplines at the controlled condition in the multicenter clinical trial provided us an opportunity to study some personal issues on the interpretation of dual-tracer PET/CT images. It was found out that with certain experience and training both radiologists and nuclear physicians are qualified to read and to achieve the similar diagnostic accuracy in PET/CT study. Making full use of modality combination and selecting right criteria seems more practical than professional back ground and personal experience in the new hybrid imaging technology, at least when novel tracer or application is concerned. In view of the limitations of the study, the conclusions need further verification by larger scale studies.
